# The Rules and Functions of Nucleocytoplasmic Shuttling Proteins

**DOI:** 10.3390/ijms19051445

**Published:** 2018-05-12

**Authors:** Xuekun Fu, Chao Liang, Fangfei Li, Luyao Wang, Xiaoqiu Wu, Aiping Lu, Guozhi Xiao, Ge Zhang

**Affiliations:** 1 Department of Biology and Guangdong Provincial Key Laboratory of Cell Microenvironment and Disease Research, Southern University of Science and Technology, Shenzhen 518055, China; ulric.fu@outlook.com; 2 Law Sau Fai Institute for Advancing Translational Medicine in Bone and Joint Diseases, School of Chinese Medicine, Hong Kong Baptist University, Hong Kong, China; liangchao512@163.com (C.L.); fayebalaba@live.com (F.L.); luyaoben@126.com (L.W.); isawu199205@gmail.com (X.W.); aipinglu@hkbu.edu.hk (A.L.); 3 Institute of Integrated Bioinfomedicine and Translational Science, School of Chinese Medicine, Hong Kong Baptist University, Hong Kong, China; 4 Institute of Precision Medicine and Innovative Drug Discovery, School of Chinese Medicine, Hong Kong Baptist University, Hong Kong, China; 5 Shenzhen Lab of Combinatorial Compounds and Targeted Drug Delivery, HKBU Institute of Research and Continuing Education, Shenzhen 518057, China; 6 Department of Orthopedic Surgery, Rush University Medical Center, Chicago, IL 60612, USA

**Keywords:** nucleocytoplasmic transport, shuttling protein, cancer, neurodegenerative diseases, leukemia, osteoporosis, fanconi anemia

## Abstract

Biological macromolecules are the basis of life activities. There is a separation of spatial dimension between DNA replication and RNA biogenesis, and protein synthesis, which is an interesting phenomenon. The former occurs in the cell nucleus, while the latter in the cytoplasm. The separation requires protein to transport across the nuclear envelope to realize a variety of biological functions. Nucleocytoplasmic transport of protein including import to the nucleus and export to the cytoplasm is a complicated process that requires involvement and interaction of many proteins. In recent years, many studies have found that proteins constantly shuttle between the cytoplasm and the nucleus. These shuttling proteins play a crucial role as transport carriers and signal transduction regulators within cells. In this review, we describe the mechanism of nucleocytoplasmic transport of shuttling proteins and summarize some important diseases related shuttling proteins.

## 1. Introduction

In eukaryotes cell, the nucleus is covered by nuclear membranes. Basic life activities such as the replication and transcription of genes occur in the nucleus, while the translation and modification of protein take place in the cytoplasm. It is of great significance for associated nucleic acid, proteins and other molecular substances to perform normal biological functions across the nuclear envelope. Biological macromolecules can transport between the nucleus and cytoplasm via the nuclear pore complexes (NPCs) embedded in the nuclear envelope which regulate the exchange of components during nucleocytoplasmic transport [1]. 

As early as 1958, Goldstein for the first time observed that certain proteins can shuttle between the nucleus and the cytoplasmic constantly. After he grafted labeled nucleus into an unlabeled *Amoeba*, he found the radioactive labeled would leave the grafted nucleus and accumulate in the unlabeled nucleus while little-labeled nucleus was found in the cytoplasm. He then postulated there should be some proteins able to shuttle between the nucleus and cytoplasm [2]. However, the above-mentioned method is hard to apply in eukaryotes and other organisms. Due to technological limitation, little progress had been made in this area until the first shuttling protein, nucleolin, was identified around 30 years later [3]. Nuclear pore complexes play a key role in nucleocytoplasmic transport whose structure and function have been an active field for 60 years. Recent advances in the NPCs structure enable us to better understand mechanisms of nucleocytoplasmic transport and identify much more shuttling proteins. Subsequently, numerous proteins involving transcription factors [4], cell proliferation regulators [5], hormone receptors [6], translation initiation factors [7], and RNA binding proteins [8] have been recognized as nucleocytoplasmic shuttling proteins.

Shuttling proteins generally have two functions. First, shuttling proteins often mediate the transportation of some special RNA and proteins across the nuclear membrane [9]. Second, shuttling proteins coordinate information transfer of life activities between the nucleus and cytoplasm [10]. Proteins with nuclear localization signals (NLSs) and/or nuclear export signals (NESs) transport across NPCs through import and export pathways, which are complicated and strictly controlled. This review discusses the mechanism that initiates protein shuttling between nucleus/cytoplasm and describes some disease-related shuttling proteins.

## 2. The Mechanism of Shuttling Proteins 

### 2.1. Structure and Function of the Nuclear Pore Complex (NPC)

**Figure 1:**
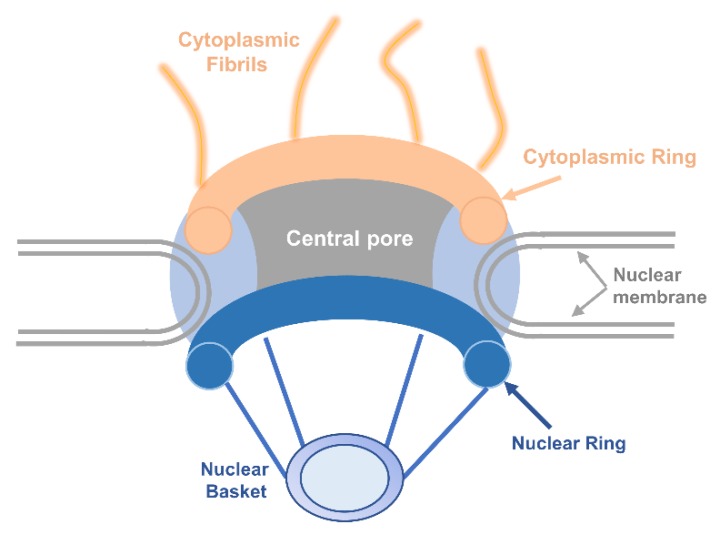
Structural characteristics of the nuclear pore complex. Schematic representation shows half of an NPC embedded in the nuclear membrane. The shuttling proteins can import or export to the nucleus through the nuclear pore complex.

The nuclear envelope in a eukaryotic cell is both a natural barrier to separate the nucleus from the cytoplasm and provides a channel for macromolecular substances to exchange through NPC embedded in the nuclear envelope [11]. There are two mechanisms for molecules to travel across the membrane. Small molecules and proteins with molecular mass smaller than around 40 kDa can passively diffuse across NPCs which have open aqueous channels with estimated 9 nm diameters for small molecules to shuttle in both directions. Other macromolecules transport in an energy-dependent manner [12]. Based on high-definition electron microscopy, reconstruction shows that architecture of all NPCs is rather conservative. Generally, NPCs have three basic elements: the nuclear basket at the length of about 100 nm that branches from the nuclear ring, the central pore, and the cytoplasmic fibrils about the length of 50 nm whose branches are from the cytoplasmic ring [13,14] (Figure 1). 

NPC is a huge protein complex that consists of around 30 different proteins collectively called nucleoporins (NUPs). Generally, the nucleoporins are divided into the following three categories. (1) Membrane NUPs: Three membrane-spanning NUP proteins contain transmembrane helices that can fasten NPC to the nuclear envelope and they can strengthen interaction between outer and inner membranes of the envelope [15]. (2) Scaffold NUPs: They serve as a linker between the membrane NUPs and NUPs with repeating amino acid sequences involving phenyalanine (F) and glycine (G) (FG-NUPs), fulfilling architectural roles and increasing the stability of the NPC [16]. (3) FG-NUPs: Characterized by repeated consensus FXFG and/or GLFG, which are the minimal domain for performing an important function in cells, most FG-NUPs reside within the central transport channel and construct the permeability barrier that can interact with transport receptors family, forming the route for nucleocytoplasmic transport [17]. 

Various models ranging from the selective phase model, virtual gate model, reversible collapse model, Karyopherin-centric model, forest model to ring cycle model at present have been suggested to explain how FG-NUPs are organized for NPC transport selectivity [18]. Among them, the “selective phase model” and the “virtual gating model” have drawn the most conspicuous attention. Both presume that FG-NUPs can construct a disordered meshwork with specific biophysical properties, attracting cargo complexes and getting through the central channel. In addition, the virtual gating model postulates that the individual affinity of FG-repeats in situ to cargo complexes is crucial to increase its possibility to go through cargo passage without the existence of much interaction among FG-repeats. Conversely, the selective phase model requires complete interaction among FG-repeats in situ to produce a sieve-like meshwork as a permeability barrier. The liquid phase separation is responsible for the selectivity of the central channel [19].

### 2.2. Transport Signals for Nuclear Import and Export

Currently, most macromolecules contain nuclear localization signals (NLSs) and nuclear export signals (NESs) for nuclear import and export. Members of karyopherin family can recognize and bind these signals of cargo proteins. Except for importin-α, importin-β1, importin-β2 and chromosome region maintenance 1 (CRM1) being well described, others of karyopherin family also have important functions in nucleocytoplasmic transport [20]. The widely known NLSs can be divided into classical NLSs including monopartite, bipartite, and non-classical NLSs. Leucine-rich NESs are one of the most widely studied signals that can be identified by CRM1. 

#### 2.2.1. Nuclear Localization Signals

**Figure 2:**
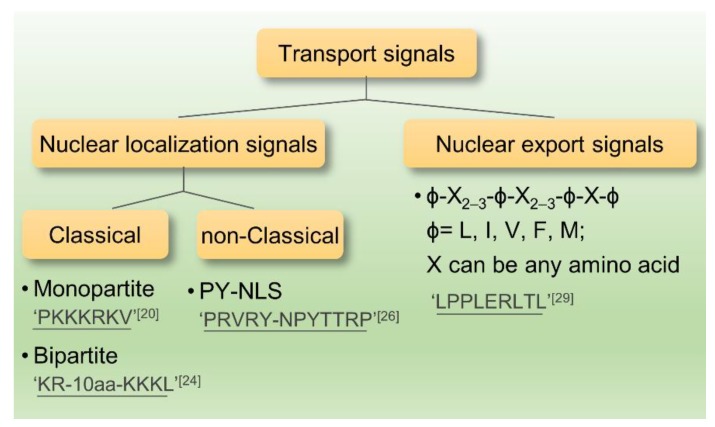
The classification and samples of nuclear transport signals.

In recent years, research on the nuclear localization signals has grown increasingly in-depth that NLSs can be classified into several categories as followed: (1) Monopartite NLSs, which were found originally in the SV40 large T antigen containing seven amino acids (PKKKRKV) [21]. It had been demonstrated that the monopartite type of NLSs are generally a short peptide comprising 4–8 amino acids and it is abundant in positively charged Lys and Arg [22]. In addition, MYC proto-oncogene (c-Myc) and nuclear factor kappa B (NF-κB) all fall into this category [23,24]. (2) Bipartite NLSs: This type of NLSs is comprised of two clusters of basic amino acids which are separated by 10–12 non-conservative amino acids. Nucleocytoplasmic protein containing (KR-10 aa-KKKL) is a typical one [25,26]. Both monopartite and bipartite NLSs pertain to the classical NLS. (3) The non-classical NLSs have no obvious feature in the amino acid sequence and the basic amino acids neither cluster alone nor enrich. Although a proline-tyrosine (PY)-NLS featuring a hydrophobic motif in N-terminal, a central arginine residue and a proline-tyrosine sequence in C-terminal was defined as a new class of non-classical NLSs, it was disordered in structure [27,28]. It is considered that the non-classical NLSs can directly bind to importin-β without the existence of importin-α [29] (Figure 2).

#### 2.2.2. Nuclear Export Signals

The currently identified NESs sequences are basically leucine-rich. First identified NES-containing protein were human immunodeficiency virus (HIV) Rev and protein inhibitor A [30,31]. Leucine-rich NES has certain consensus sequence: ϕ-X_2–3_-ϕ-X_2–3_-ϕ-X-ϕ (ϕ = L, I, V, F, M; X can be any amino acid). Larger hydrophobic amino acids, such as leucine and isoleucine, are crucial to sustaining the spatial structure. There exists an important feature of leucine-rich patterns; most the amino acids in the hydrophobic amino acids are negatively charged [32,33]. The process of leucine-rich NES-regulated nuclear export requires energy and has a saturation effect, suggesting that some specific receptors are essential to identify this NES and export the protein to the cytoplasm by active transport [34]. 

The study showed that as a relatively conserved protein, the nuclear transporter CRM1/exportin-1(CRM1, chromosome region maintenance 1) is a leucine-rich receptor [35]. Sequence alignments indicate that the sequence homology in human and yeast reaches 42–50% [36]. The protein structure is key to the function of NES. For instance, NES, which is isolated from HIV Rev protein, has significantly lower binding ability to CRM1 than full-length Rev protein and can bind to CRM1 only when the protein has specific structure [33,37] (Figure 2).

### 2.3. Transport Receptor Family

Nuclear transport receptors (NTRs), also known as karyopherins family, are crucial to the active transport of the proteins. The transient interaction of NTRs with the NPC complex facilitates protein transports passage through the nuclear envelope. In addition, the interaction is regulated by GTPase Ran. Nuclear transport receptors mainly span two family, karyopherin-β and karyopherin-α, also known as importin-α [20,38].

Importin-α acts as an adaptor during nuclear import of proteins, recognizing and ligating the protein between importin-β and cargo proteins. In mammalian cells, the importin-α family consists of six members, namely importin-α1/Rch1, importin-α3/Qip1, importin-α4/hSRP1γ, importin-α5/hSRP1, importin-α6 and importin-α7 [39,40]. The expression patterns of this family are associated with the species and differentiation status of the cells. Different importin-α have their unique NLS binding profiles. Importin-α can precisely recognizes cargo proteins by virtue of classical NLS and it also has an importin-β binding (IBB) domain [40,41]. 

The karyopherin-β family is a wide range of nuclear transport receptors in eukaryotes and there are at least 20 in human cells [42]. Karyopherin-β, with a heat-repeated fold, can provide a binding site for protein interaction [43]. Most karyopherin-β proteins can directly recognize and bind the nuclear protein signal (NLS) or nuclear export signal (NES) to regulate protein import and export, but some receptors cannot recognize the substrate without special adaptor proteins [20]. For example, importin-β1 needs the importin-α to recognize the classical NLS-containing protein, and then importin-β1 binds to the importin-α through the N-terminal IBB so that it participates in the subsequent interaction with nucleoporin of NPC [44].

The exportins, belonging to the karyopherin-β, can recognize nuclear export signals, leucine-rich NES in particular [12]. CRM1, one of the exportin proteins, is responsible for transporting many proteins from the nucleus into the cytoplasm with or without adapter proteins [33,45,46]. Apart from CRM1, there are many studies on other export receptors. It is reported that at least exportin-t and exportin 5 can help several types of RNA export from the nucleus. The former enables tRNA to export, while the latter mediates double strand RNA and pre-microRNA to the cytoplasm whose RNA hairpin structure is recognized as an NES [47,48,49]. Exportin 4 is responsible for eukaryotic translation initiation factor 5A (eIF5A) and mothers against decapentaplegic homolog 3 (Smad3) export [50]. Exportin cellular apoptosis susceptibility protein (CAS) is of great significance because it can transport import-α from the nucleus to the cytoplasm for a new cycle of protein import [51].

NTRs regulate the nucleocytoplasmic transportation of important cellular components via actively transporting their cargo across the nuclear envelope. Various human diseases occur as a result of alternations of transport system pathways caused by the mutations of the nuclear transport system. Identification of more NTR-specific cargoes spectrum can help us to better understand the nucleocytoplasmic transport system. By virtue of SILAC-Tp (stable isotope labeling with amino acids in cell culture), the import cargoes of all 12 NTRs including 10 imports and two bi-directional receptors were identified [52]. The other advanced method, BioID that used proximity ligation and mass spectrometry successfully identified the specific interactions between NTRs and over 1200 proteins in which many fractions were the first experimental evidence. The study also described the potential transport pathways of most cargos [53]. These studies strongly demonstrate that individual NTRs can play multiple roles because of the interaction between large protein complexes, functionally related proteins involved in different cellular processes and specific NTRs. 

### 2.4. The Ran System

Ran protein is a small Ras-like GTPase and can switch between GDP binding and GTP binding [54]. Nucleocytoplasmic transport of proteins is regulated by Ran and needs GTP hydrolysis [20]. Ran can slowly hydrolyze RanGTP into RanGDP with the help of RanGTP-activating protein (RanGAP) and cofactor RanBP (Ran-binding protein). Then, RanGDP transforms into RanGTP after its interaction with RanGEF (Ran guanine exchange factor) [55,56]. RanGAP is mainly concentrated in the cytoplasm while RanGEF is in the nucleus, so the concentration of the RanGTP in the nucleus is higher than in the cytoplasm [57]. Concentration gradient provides a guarantee for nuclear import and export [58]. RanGTP directly binds importin-β, but not importin-α and once combined, the import complexes would release the cargo protein. In contrast, the export complexes are formed by association with RanGTP [59].

### 2.5. Nuclear Import of Proteins 

**Figure 3:**
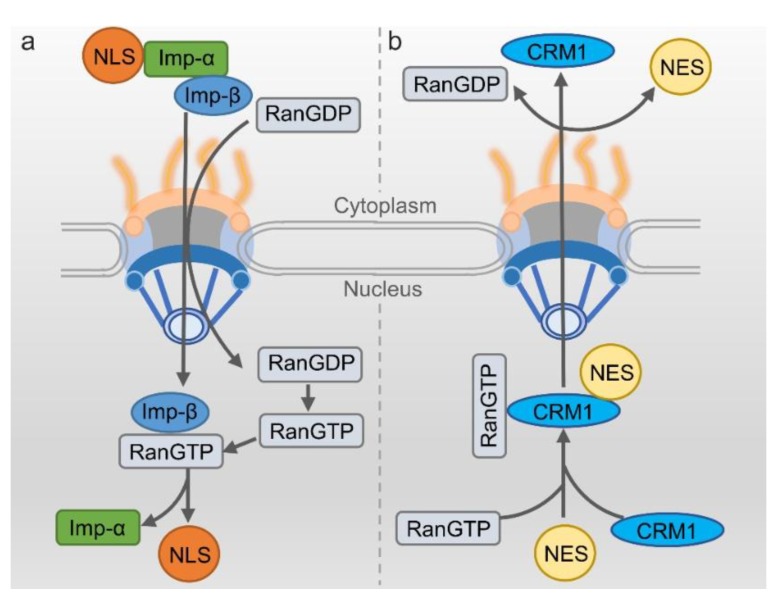
Simplified nucleocytoplasmic transport process of proteins. (**a**) Nuclear Import: The protein with the nuclear localization sequence (NLS) transporting into the nucleus is mediated by the importin-α/importin-β1 heterodimer (designed as imp-α and imp-β). Nuclear import of NLS cargo through binding to importin-β only is not shown. (**b**) Nuclear Export: The export of protein containing nuclear export sequence (NES) from nuclear is mediated by CRM1 dependent on Ran system.

The classical nuclear import process highly relies on nuclear import receptor importin α/β. This transport mechanism is relatively clear [60]. To initiate the process, firstly importin- α recognizes the NLS-containing cargo protein and then heterodimerizes with importin β. The complex of cargo/importin-α/importin-β then localize to the nuclear envelope. Importin-β can interact with nucleoporins, mediating the translocation through NPC. After the import complex is translocated into the nucleus, RanGTP would bind to importin-β and change its structure, dissociating the import complex and releasing the cargo protein into the nucleus. Importin-β/RanGTP can directly transport back to the cytoplasm, but importin-α needs the assistance of nuclear exporter to go back such as CAS [61]. Another type of protein goes into the nucleus without the involvement of importin-α through direct recognition of the importin-β2 mediating into the nucleus. In this mechanism, most target proteins have NLS for recognition and being bound by importin-β2, but these NLSs lack regularity in the amino acid sequence composition [29] (Figure 3a).

### 2.6. Nuclear Export of Proteins

The best characterized mechanism of nuclear export needs the exportin CRM1 to bind the leucine-rich NES-containing cargo [35]. During the process of nuclear export, CRM1 firstly binds with RanGTP in the nucleus. With the help of RanBP and RanGEF, the affinity of CRM1 with cargo protein is improved. The RanGTP/exportin/cargo complex transport to the cytoplasm through NPC [62,63]. The GTP would be hydrolyzed when the complex of RanGTP/exportin/cargo binds with RanGAP, leading to the detachment of the complex. Then the cargo protein would be released into the cytoplasm [64]. RanGTP and the CRM1 return to the cell nucleus via the NPC to participate in a new round of nuclear export (Figure 3b).

## 3. Shuttling Proteins and Diseases

### 3.1. Shuttling Proteins in Cancer

**Figure 4:**
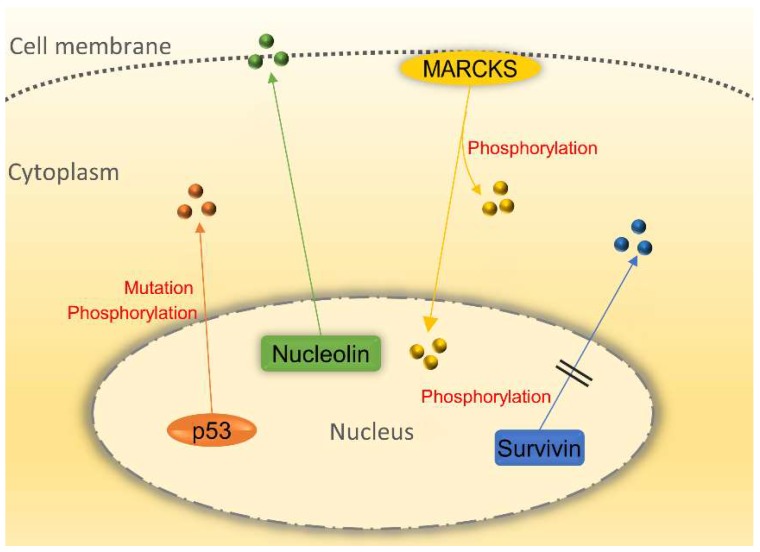
Nucleocytoplasmic transport of shuttling proteins involving in cancer development. p53 localizes in the cytoplasm of cancer cells instead of the nucleus due to its phosphorylation and mutation while losing its basic function. The inactivation of survivin’s NES by phosphorylation could promote nuclear survivin accumulation and the susceptibility of cancer cells to chemotherapy and radiation treatment. Nucleolin shuttles from the nucleus to the membrane to facilitate tumor growth. Myristoylated alanine-rich C kinase substrate (MARCKS) can be translocated from the cell membrane to the cytoplasm and nucleus to perform a function. All circles represent the corresponding proteins.

A series of factors such as chemicals, radiation, diet, infection, and heredity have been reported to involve in the pathogenesis of cancer. To date, an increasing number of shuttling proteins have been found to be associated with cancer (Figure 4).

p53 is a main regulator of the cell cycle at the G1/S regulation point and tumor suppressor. DNA repair protein can be activated by p53 when DNA is damaged. Therefore, p53 whose mutant form has been found in most of the cancer cells is the key to arrest the damaged cell cycle [65]. Subcellular localization of p53 is closely related to the onset of cancer. p53 mutant is the most common way for p53 function silencing, but it has been discovered that wild-type p53 is localized in the cytoplasm of tumor cells [66]. This phenomenon implicates that p53 can be inactivated when localizing in the cytoplasm because only when p53 enters the nucleus can it work. Thus, it may be a good way to prevent tumor growth by blocking the translocation of p53 from the nucleus to cytoplasm. Post-transcriptional modification of p53 protein is crucial for its nucleocytoplasmic shuttling function. Many studies have shown that the inhibition of Thr-55 phosphorylation of p53 protein not only can prevent the p53 protein from CRM binding and nucleocytoplasmic transportation but also can enhance the sensitivity of cancer cells to DNA damage [67,68]. The study provided a new way to investigate the abnormal localization of p53 in the cytoplasm. Squamous cell carcinoma includes head and neck squamous cell carcinoma (HNSCC), can spread over many body organs including the mouth and throat. It is a kind of fatal cancer which is hard to prognose [69]. The main problem of HNSCC treatment is cisplatin drug resistant. By evaluating nucleocytoplasmic distribution, research has demonstrated that p53 nuclear localization signal will be lost in HNSCC cell lines. The reason for is that the nuclear localization signal of p53 has mutated, thus keeping p53 from shuttling into the nucleus and reducing its sensitivity to cisplatin medication [70]. Moreover, p53 can regulate transport receptor proteins. Tanani et al. showed that the CAS, an exportin that can transport importin-α back to the cytoplasm, was significantly up-regulated in Hepatocellular carcinoma (HCC) patient. The CAS/importin-α transport cycle is of critical importance for tumor cell survival. Meanwhile, p53 can mediate CAS repression, which could be used as a novel way to regulate tumor cell growth in hepatocarcinogenesis [71]. 

Survivin is bifunctional when shuttling between the nucleus and the cytoplasm. It takes part in cell division regulation in the nucleus and it controls apoptotic pathways in the cytoplasm [72,73]. Survivin contains conserved CRM1-dependent leucine-rich NES and a non-classical NLS sequence in the C-terminus. The intracellular localization is essential for survivin to promote tumor development. NES-deficient survivin results in mitotic arrest and polyploidy in Hela cells. Cancer patients have a higher chance of survival because of survivin’s localization in the nucleus makes cancer cells more susceptible to chemotherapy and radiation treatment [74]. In those head and neck cancer patients with favorable prognostic, mutation analysis in HNSCC biopsies found that survivin mutation in c.278T>C (p.Phe93Ser) can inactivate survivin’s NES, leading to a dominant nuclear accumulation, and can moderate survivin’s cytoprotective activity because it can curb chemoradiation induced apoptosis. This study has clearly proven that genetic inactivation of survivin’s NES could give rise to nuclear survivin accumulation and enhance therapeutic effect in cancer patients [75]. More recently, it has been found that survivin can regulate tumor microenvironment through secreting to the extracellular space in the form of exosome as vesicles to transport proteins and RNAs. Survivin derived from Serum and plasma has been detected in manifold cancers. Stress can induce survivin release along with exosome in Hela cells. Exosomal survivin may be used as a marker for early detection and diagnosis of prostate cancer [76]. Although exosomal survivin plays an important role in cancer progression, the relationship between intracellular shuttling of survivin and exosomal survivin still needs further exploration.

Myristoylated alanine-rich C kinase substrate (MARCKS), a main substrate of protein kinase C (PKC), plays an essential role in cancer development and progression. MARCKS is usually located on the cell membrane to shuttle between the cytoplasm and the cell membrane. Located in the plasma membrane, unphosphorylated MARCKS would be translocated to the cytoplasm after its phosphorylation by PKC [77,78]. It has been found that phosphor-MARCKS is highly expressed in melanoma cells. As a result, several MARCKS are released from the membrane into the cytoplasm to promote actin reorganization, cell motility and metastatic potential of melanoma cells [79]. Chen et al. showed that an increase of phospho-MARCKS can activate lung cancer cells growth and there exists a high correlation between the phospho-MARCAS and lower survival chance for lung cancer patients, suggesting that phospho-MARCAS, which is transferred to the cytoplasm, leads to the detachment of phosphatidylinositol-4,5-bisphosphate (PIP2) from the membrane. PIP2 can act as a substrate for phosphatidylinositol-4,5-bisphosphate 3-kinase (PI3K) which activates the serine/threonine protein kinase (AKT) pathway, eventually promoting cancer cells growth [80]. MARCKS cannot only shuttle between the cytoplasm and the membrane but also transfer to the nucleus. Studies have proved that the effector domain of MARCKS can be recognized as a nuclear localization signal which enables MARCKS to enter the nucleus in glioblastoma cells. In the nucleus, MARCKS regulated PIP2 levels and fulfil many cell function [81]. 

Nucleolin is a non-ribosomal protein and the first identified shuttling protein [3]. It is most abundant in the nucleolus and plays a crucial role in polymeraseI transcription [82]. In addition, it locates outside the nucleolus and cytoplasm, and even moves to the cell membrane [83]. Studies have shown that the cell membrane of Nucleolin is strongly correlated with the development of cancer. In the primary glioma cells, the level of glycosylated nucleolin on the plasma membrane of cells is proportion to the level of human malignant tumor [84]. HB-19 pseudopeptide, a specific antagonist targeting C-terminal of nucleolin of cell surface, can be used to delay tumor growth in nude mice. Meanwhile, the tumorigenic potential of the TIII cells (a melanoma cell line) derived from melanoma tumor would be impaired [85]. Nucleolin on the surface of the cells takes effect when it binds to other proteins. Christian et al. identified that by affinity chromatography, nucleolin can bind to F3 peptide that is tumor-homing peptide on the MDA-MB-435 cell (a breast cancer cell line) surface. Nucleolin can turn internalized F3 peptide into tumor cells to promote tumor angiogenesis. This study provided strong evidence that shuttling nucleolin can serve as a novel vascular marker [86]. Nucleolin also involves in miRNAs biogenesis, a class of non-coding single-stranded RNAs. The research found that 4LB5 as a fully engineered human single-chain fragment can be bound to nucleolin on the cell surface of breast cancer cell lines. It can abolish nucleolin-dependent biogenesis of miR-21, -221 and -222, thus inducing apoptosis of cancer cells and reducing tumor volume in vivo. This study again has proven that shuttling nucleolin between the nucleus and membrane plays a decisive role in cancer development [87].

### 3.2. Shuttling Proteins in Neurodegenerative Diseases

Neurodegeneration is a gradual loss of function and the death of neurons. Amyotrophic lateral sclerosis, Alzheimer’s disease, Parkinson’s disease and Huntington’s disease are the main neurodegenerative diseases [88]. The pathogenesis is rather complicated, but basically it is related to misfolded proteins aggregation [89,90]. Misfolded proteins have features of being refolded or being sequestered to different cellular sites. However, some studies have found that abnormal subcellular localization of proteins is related to the incidence of diseases [91]. Diseases such as neurodegenerative diseases occur when misfolded proteins are apt to assemble as toxic aggregate. Woerner et al. demonstrated that aggregates in different intracellular localization produce cytotoxicity. In the study, an artificial β-sheet protein is used to simulate properties of amyloid aggregates and a soluble α-helical bundle is utilized as a non-toxic control. Then, nuclear localization signals and nuclear export signals are attached to proteins to identify the source cellular compartment of toxicity. Stained with the amyloid-specific dye, it turns out that cytoplasmic β-sheet proteins had a higher level of toxicity than its nuclear counterparts. It also found aggregates interacted with NPM1, an abundant negatively-charged protein in the nucleus, which can act as chaperone to inhibit the potential toxicity of β-sheet protein aggregates. Moreover, the study also proved that THOC2, a subunit of a messenger RNA export complex, was mislocalized into the cytoplasm and interacted with cytoplasmic β-sheet protein aggregates. The reason misfolded protein aggregation in the cytoplasm is toxic is that the nuclear import and export are inhibited [92]. Their research is meaningful because it has strongly proved that cytoplasmic aggregates and nuclear import-export impairment, could be common feature for many human neurodegenerative diseases.

Alzheimer’s disease (AD), a chronic neurodegenerative disease of neurological disorder, is attributed to the degeneration of the neuronal population in the hippocampus. The pathogenesis of this disease is still under investigation. Ogawa et al. used hippocampal tissues from AD patients to conduct immunohistochemistry and found phosphorylated H3, an important modification in chromosome compaction during cell division, increased in several AD patients’ neuron. More importantly, phosphorylated H3 was mislocalized in the neuronal cytoplasm instead of the nucleus [93]. Similar observations were made in experiments on Huntington’s disease patients [94]. Oxidative stress is another common pathogenesis of Alzheimer’s disease. Damage occurs in the early stage of the disease and can accelerate the progression of the disease. The nuclear factor E2-related factor2 (Nrf2) is a transcription factor which can regulate the levels of antioxidant proteins by entering into the nucleus, and now it is reported to be related to AD, especially its subcellular localization. Ramsey et al. found that the expression pattern of Nrf2 changed in hippocampi tissue of AD patients by studying their brain. Moreover, it proved that Nrf2 was mislocalized in the cytoplasm rather than the nucleus where it exerted antioxidative stress to AD patients, implicating that the course of Nrf2 import to the nucleus was impaired during AD progression [95]. The aberrant localization of a protein in diseased cells may result from the problematic transport receptor. That protein entry to the nucleus is mediated by importin. Compared with control patients, importin-α was found specifically localize to Hirano bodies in the cytoplasm of the hippocampus of AD patients. Hirano bodies are cytoplasmic inclusion whose formation could be found in the neurodegenerative diseases. The abnormal localization of importin-α1 hindered its transportation of NLS-containing protein to the nucleus and therefore brought about a variety of cell dysregulation [96]. The present study provides plenty of evidence that transport receptor family for protein nucleocytoplasmic shuttling may be involved in neurodegenerative disease progression.

Amyotrophic lateral sclerosis (ALS), also known as Lou Gehrig’s disease, is featured with muscle stiffness and muscle twitching. It leads to the death of neurons dominating voluntary muscles. Little is known about the pathogenesis of ALS and research has found that about 10% of the cases is attributed to heredity [97]. There also exists dysfunctional nucleocytoplasmic transport for the disease. One of the most outstanding pathological features in ALS is the redistribution of nuclear proteins to the cytoplasm. TAR (RNA regulatory element) DNA-binding protein 43 (TDP-43) is a conserved nuclear ribonucleoprotein (hnRNP) that can regulate transcription and alternative splicing. The immunofluorescence study on the cellular localization demonstrated that endogenous TDP-43 was mainly localized in the nucleus and that TDP-43 had the ability to shuttle between the nucleus and cytoplasm. TDP-43 C-terminal tail was essential for its location in cellular targeting [98]. It was found in ALS that there was aberrant accumulation of TDP-43 in neuronal cytoplasmic inclusions. There were a small number of TDP-43 in the cytoplasm under physiological condition, but the pathology accumulation of cytoplasmic TDP-43 increased dramatically in the ALS patients [99]. Another protein, FUS, an RNA binding protein, shares some features with TDP-43. Nucleocytoplasmic transport of FUS would be disrupted by mutation in its NLS or C-terminal truncation and FUS would be redistributed to the cytoplasm, which is closely related to ALS [100]. The mislocalization of TDP-43 and FUS to the cytoplasm in ALS implies that impaired nuclear import plays a critical role in ALS. The importance of nuclear transport in the pathogenesis of the disease was strongly proven by the fact that the level of nuclear import factors such as importin-β1 and importin-β2 is reduced and mislocalized in tissues of ALS patients [101].

### 3.3. Shuttling Proteins in Other Diseases

#### 3.3.1. Shuttling Proteins in Acute Myeloid Leukemia

Acute myeloid leukemia (AML) is a malignant disease of myeloid hematopoietic cells, characterized by abnormal proliferation of bone marrow and blood cells. Nucleophosmin (NPM) is a nucleocytoplasmic shuttling protein with prominent nuclear localization. One-third of ACL is thought to be related to aberrant localization of NPM in the cytoplasm. The study of subcellular localization of NPM in bone marrow-biopsy specimens with AML confirmed that the mutations of NPM could alter the protein at C-terminal causing its cytoplasmic localization in cells [102]. Similar conclusion could be drawn in childhood acute myelogenous leukemia patients [103]. NPM regulates cell proliferation and stimulates cell survival. Further molecular basis study on NPM demonstrated that mutation of four amino acids in C-terminus could form a nuclear export signal. This gain-of-function of NPM created a new functional NES, causing its largely aberrant localization in the cytoplasm, which might be regarded as the mechanism of leukemogenesis and a target for therapy [104].

#### 3.3.2. Shuttling Proteins in Osteoporosis

Osteoporosis, one of the most common aging-related diseases, is characterized by low bone mass, damaged microstructure, fragile bone and greater vulnerability to fracture. Along with the increase of the aged population, osteoporosis has become a global public health problem. Bone remodeling occurs throughout life. It is essential for successful bone remodeling to have an exact coupling between osteoclast mediated bone resorption and osteoblast-mediated bone formation. When bone formation is damaged or resorption enhanced, osteoporosis occurs. Significant progress has been achieved in the pathogenesis of osteoporosis through the investigation of bone formation and resorption. Localized at cell adhesion plaques, Cas-interacting zinc finger protein (CIZ) is a nucleocytoplasmic shuttling transcriptional repressor. Some studies have demonstrated that CIZ plays a key role in bone formation. In CIZ-deficient mice, bone mass and bone volume were enhanced. Bone marrow cells derived from the CIZ-deficient mice can get higher alkaline phosphatase (ALP) activity and mineralized ability compared to wild-type cells. Moreover, CIZ can regulate mechanical stress. Unloading through tail suspension can reduce bone mass in wild-type mice remarkably, but CIZ-deficient mice reversed the bone mass loss [105,106]. These studies only confirm the relationship between expression of shutting proteins and bone formation but lack a specific description of proteins localization. Chen et al. found that RAN binding protein 3 like (RanBP3L) can regulate Smad1/5/8 to enter the nucleus in a Ran-dependent manner. It is important for BMP signaling to induce osteogenesis with nucleocytoplasmic shuttling of Smad1/5/8. Knockdown of RanBP3L can promote mesenchymal stem cells differentiation [107].

#### 3.3.3. Shuttling Proteins in Fanconi Anemia

Fanconi anemia (FA) is a rare genetic disease whose response to DNA damage is impaired. It features congenital defects, bone marrow failure, and developmental abnormality. Quite a few patients with FA will develop cancer. FA is an autosomal recessive genetic disorder which is caused by bi-allelic mutation of any one of sixteen genes. Proteins produced by these genes can repair DNA damage-dependent on FA-BRCA pathway. The monoubiquitination of Fanconi anemia complementation group D2 (FANCD2) and Fanconi anemia complementation group I (FANCI) protein can turn on this pathway by localizing the chromatin region. Research has found that terminal 58 amino acid is the NLS of FANCD2. More importantly, mutations of these sequences are unable to repair the DNA interstrand crosslinks (ICLs) sensitivity in FA-D2 patient cells, which reveals a novel relationship between the nuclear import of FANCD2 and FA disease [108].

## 4. Perspective and Conclusions

It is essential for proteins to correctly regulate cell growth and death with their appropriate and accurate localization. Proteins that can transport between the nucleus and the cytoplasm are called shuttling proteins or nucleocytoplasmic shuttling proteins. Nucleocytoplasmic shuttling is extremely complicated and tightly regulated. Many important proteins take part in the process such as NUPs in nuclear pore complex, nuclear transport receptors, and Ran system. NPC can produce a natural barrier between the nucleus and cytoplasm to select proteins transport when proteins shuttle across nuclear envelop. The transport receptor families, including importin and exportin families, play a crucial role in nucleocytoplasmic shuttling. They can recognize and bind either the nuclear localization signal or nuclear export signal within the cargo proteins to generate transport complex in existence of Ran system. Then, the transport complex interacts with the nucleoporin of NPC. It briefly describes the whole process of nucleocytoplasmic transport with each step accompanied by numerous protein interactions and dissociations.

Interactions regulation will affect correct subcellular localization of target proteins and their biological function. Dysregulation of shuttling proteins leads to mislocalization of proteins, which is relevant to several diseases. It has been reported that more and more shuttling proteins such as transcription factors, cell cycle regulators and other functional proteins are also found in a variety of diseases. The feature of abnormal protein localization in cells has attracted our attention. We should not only consider the expression levels of proteins but also detect the localization of the protein in the pathology condition of cells. 

Given the relationship between shuttling proteins and diseases, results from present research on the nucleocytoplasmic transport can be applied to diseases diagnosis and new drug design, because every biomolecule involved in the shuttling process is a potential target. Currently, some compounds have been used as CRM or importin α/β inhibitors to regulate subcellular localization of proteins in diseases [109,110]. With further study on the mechanism of protein transport, we will have a better understanding of the connection between shuttling proteins and pathogenesis to find more novel targets for diseases treatment.
